# Survival of rural telehealth services post‐pandemic in Australia: A call to retain the gains in the ‘new normal’

**DOI:** 10.1111/ajr.12877

**Published:** 2022-05-25

**Authors:** Lisa A. Caffery, Olav T. Muurlink, Andrew W. Taylor‐Robinson

**Affiliations:** ^1^ School of Business and Law Central Queensland University Emerald Queensland Australia; ^2^ School of Business and Law Central Queensland University Brisbane Queensland Australia; ^3^ College of Health Sciences Vin University Hanoi Vietnam; ^4^ Center for Global Health University of Pennsylvania Philadelphia Pennsylvania USA; ^5^ School of Health, Medical & Applied Sciences Central Queensland University Brisbane Queensland Australia

**Keywords:** COVID‐19, rural health policy, telehealth, urban paternalism, virtual health care

## Abstract

**Aim:**

COVID‐19 rapidly transformed how Australians access health care services. This paper considers how the inability for urban patients to access in‐person care expediated the introduction of virtual solutions in health service delivery thus creating a new access paradigm for rural and remote Australians.

**Context:**

‘Physical distancing’ is a phrase synonymous with public health responses to COVID‐19 in Australia, but distance is a decades‐long problem for rural health access. Counterintuitively, the pandemic and associated restrictions on mobility have reduced *in real terms* the distance from, and therefore the time taken to access, critical public services. ‘Lockdowns’ have *unlocked* health access for rural and remote Australians in ways that had been rejected prior to 2020. The pandemic has disrupted traditional delivery models and allowed the piloting of novel solutions, at the same time as stress‐testing current delivery systems. In the process, it has laid bare a myopia we term ‘urban paternalism’ in understanding and delivering rural health.

**Approach:**

This commentary outlines how the COVID‐19 operating environment has challenged traditional urban‐dominated policy thinking about virtual health care delivery and how greater availability of telehealth appointments goes some way to reducing the health access gap for rural and remote Australians.

**Conclusion:**

Australian Commonwealth Government policy changes to expand the Medical Benefit Scheme (MBS) to include telephone or online health consultations are a positive initiative towards supporting Australians through the ongoing public health crisis and have also created access parity for some rural and remote patients. Although initially announced as a temporary COVID‐19 measure in March 2020, telehealth has now become a permanent feature of the Medicare landscape. This significant public health reform has paved the way for a more flexible and inclusive universal health care system but, more importantly, taken much needed steps towards improving access to primary health care for patients in rural and remote areas. Now the question is: Can the health care system integrate this virtual model of delivery into ‘business as usual’ to ensure the long‐term sustainability of telehealth services to rural and remote Australia?

**What is already known about this subject:**

- Physical access to timely, reliable and quality clinical care is not guaranteed for people living in rural and remote communities in Australia
- Digital health technology was not widely employed in rural and remote Australia pre‐pandemic

**What this study adds:**

- The pandemic had unintended positive consequences in forcing greater flexibility into health delivery systems
- Approaches that had previously been resisted, were embraced, and resulted in the levelling of some health access deficits in rural and regional Australia
- Now that measures to increase flexibility in delivery of health beyond the traditional face‐to‐face model have been proven effective, further reform of primary health care delivery needs to be investigated and implemented

## 1 INTRODUCTION

Physical distancing and lockdowns became part of the lexicon of coronavirus disease 2019 (COVID‐19) and public health in 2020.^1^ These terms are ironic to rural Australians who are no strangers to overcoming vast distances just to visit their general practitioner (GP) or attend a specialist appointment.^2^, ^3^ However, for those living in well‐resourced metropolitan areas an inability to easily access vital health services was a largely foreign concept—that is, until the COVID‐19 pandemic led to sudden access difficulties in urban regions in 2020.^4^ The emergence of a global pandemic has extended the experience of exclusion and isolation from the rural health system to urban areas. COVID‐19 has created an environment where, for the first time since the introduction of Medibank (Australia's universal health insurance scheme now renamed Medicare) in the 1970s (prior to which around a third of health consumers experienced difficulty in obtaining affordable health care), access to health care became problematic in Australia's major urban centres.^5^

In this paper we define ‘access’ in terms of timeliness and affordability for the health consumer. The consequence of poor health access is the unequal distribution of opportunity and lowered health outcomes.^6^ In other words, access is a non‐medical determinant that can either enable or hinder good health.^6^ Entry points into the public health system during the pandemic have become limited due to emergency facility specialisation (certain hospitals becoming COVID‐19‐focused), staff shortages (with COVID‐19‐positive staff isolated) or closure of face‐to‐face options. Periodically, elective surgery has been cancelled and metro multispecialty hospitals have experienced intermittent lockdown.^7^ Urban health consumers are consciously making the decision to delay medical treatment due to travel restrictions and safety concerns; at other times, the decision has been taken out of their hands. These circumstances are commonplace and longstanding for rural and remote patients, but represent a confronting new reality for city dwellers. The pandemic has challenged traditional face‐to‐face care models and imposed a step‐change in health delivery systems that was borne out of necessity. An inability for GPs (the focus of this commentary) to provide in‐person care to urban patients under lockdown conditions has cast a sharp spotlight on health access issues across Australia.

## 2 URBAN BIAS

An unconscious form of metropolitan bias has dominated the Australian health landscape for decades.^8^ As far back as 1955, it was observed that health policy is often instigated in urban settings, by urban dwellers with an urban bias.^9^ Health services and resources are heavily concentrated in highly visible settings such as city hospitals, while health practitioners prefer living in metropolitan areas.^10^ Health access voids are a commonplace experience for rural Australians but less so for their metropolitan peers. An ever‐persistent rural health access gap seems to escape the attention of politicians and policy‐makers in much the same way that rural and remote settlements also tend to exist beyond the public view.^6^ In other words, rural Australia is not in plain sight to Australia's highly concentrated urban centres where the overwhelming majority of its political and bureaucratic policy‐makers are based; hence, rural health needs can be overlooked in policy and planning discourse.^6^

In fact, Australia is one of the most urban‐developed nations in the world.^11^ This creates an environment in which we believe *urban paternalism* flourishes. Paternalism is characterised by people in authority regulating policy or practices in their own interest. The Macquarie Dictionary defines paternalism as ‘the principle or practice, on the part of a government or of any body or person in authority, of managing or regulating the affairs of a country or community, or of individuals, in the manner of a father dealing with his children, especially to the extent that individual rights are abrogated, such as freedom of choice and individual responsibility’.^12^ Thus, urban paternalism within a health care context suggests policy and practice is inevitably framed from an urban standpoint, whereby the rural case to which it is compared is seen as the exception. As Sher and Sher put it, the ‘paternalistic presumptions of capital city‐based policy‐makers far removed from the rural scene’^13^ guide the national approach. History shows that rural and remote disadvantage has never provided sufficient impetus to significantly change health care practices and to accommodate the access needs of rural Australians.^14^, ^15^, ^16^ The public health response to COVID‐19 has prompted urban‐based decision‐makers to reach a direct understanding of what disparities in access mean to Australians living outside urban areas.

## 3 SHIFTING PERSPECTIVES

An unexpected outcome from COVID‐19 was the rapid expansion of telehealth. Telehealth services include virtual care via ‘video communication, remote consultation, telephonic videos, remote monitoring, provider‐to‐provider communication, apps and Web‐based platforms’.^17^ The European Code of Practice for Telehealth Services defines telehealth as ‘the means by which technologies and related services concerned with health and well‐being are accessed by people or provided for them irrespective of location’.^18^ Put simply, telehealth is ‘health care carried out at a distance’.^19^ Although virtual models of care have been available in Australia for decades, widespread implementation continued to lag despite growing evidence supporting its clinical acceptability, appropriateness and feasibility.^19^ For example, the Australian Commonwealth Government has funded the healthdirect Video Call service since 2014, which is a video consultation platform purpose‐built for use in a health setting.^20^ This system is promoted as a flexible, efficient and secure alternative to in‐person consultations.^20^ However, pre‐pandemic many clinicians regarded telehealth as an inferior substitute for in‐person care,^21^ when the notion of wholesale transition to remote telehealth consultations was inconceivable. Known barriers to widespread sector uptake of telehealth included health care provider's data security and privacy concerns, an unwillingness to change models of care, technological issues and set‐up costs, administrative challenges, and limited professional skills and confidence to get online.^21^ There were also financial barriers for health service providers relating to payment incentives, billing and funding.^17^ Reimbursement for services through the Medical Benefit Schedule (MBS) was not universally available in Australia prior to the pandemic.^22^ However, meeting the unprecedented demands of the COVID‐19 environment has challenged traditional urban thinking about the merits of virtual health care delivery, because of overcrowded hospital emergency departments, high patient demand for GP services and constrained capacity across the health system. The need to flatten the COVID‐19 infection curve through community‐wide lockdowns and the expansion of digital health care in turn unlocked health access for rural and remote Australians in ways that were considered impossible prior to 2020.

## 4 RAPID ADOPTION

Telehealth had been used relatively sparingly in rural and remote health delivery where in‐person delivery of health was impracticable.^23^ Research undertaken by the Australian Medical Association prior to the COVID‐19 pandemic suggested that telehealth alone was not a viable solution to reduce rural health disparities but had the potential to complement existing services and improve access.^24^ Nevertheless, pandemic lockdowns, home quarantine and physical distancing practices rapidly propelled digital health care into conventional medicine. Telehealth became a standard consultation forum for health professionals working with urban clientele, because it limits person‐to‐person contact and enables health professionals to work from isolation.^25^ Personal safety (principally from contracting SARS‐CoV‐2 infection) was commonly cited as the main reason for this swift adoption.^26^ However, disruption to face‐to‐face services and the unexpected cessation of income streams were also driving factors that compelled health providers to pivot to remote servicing practically overnight.^27^ This dramatic shift was facilitated by the Royal Australian College of General Practitioners (RACGP) when, in March 2020, it implored the Australian Commonwealth Government to introduce a time‐limited Medicare rebate for telehealth consultations to allow GPs to interact with their patients irrespective of location.^28^ Subsequently, towards the end of 2020 the RACGP reported that 97% of Australian GPs were using telehealth to provide patient care during the pandemic.^29^ This was a fivefold increase from the previous year. At least temporarily, telehealth became mainstream.^30^ Furthermore, a trend analysis of Medicare data showed a 50% reduction in in‐person mental health consultations between January 2019 and June 2020 and a steep increase in the overall uptake of telehealth consultations associated with mental health providers.^26^, ^30^

Regardless of the motivations, this pandemic‐driven paradigm shift to large‐scale uptake of remote consultations has led to tangible health access gains, particularly in rural and remote settings. First driven by this emergency, customisable software systems and off‐the‐shelf technologies are now embraced and breaking down access barriers.^25^ Consumer‐facing audio visual applications such as FaceTime, Skype, Zoom, and Google Meet and Chat were deployed in Australia, the US and UK to provide care at a distance.^31^ The similar online platforms WeChat, Tencent and Hotline were actively utilised to provide telehealth services in China to connect with patients during the COVID‐19 pandemic.^32^ Telehealth has transitioned to being regarded as convenient and efficient by clinicians and patients alike.^20^ Virtual consultations obviate the need to travel, which is especially important to people living in rural, regional and remote areas. Seemingly formidable obstacles around the interoperability of technology prior to the pandemic have evaporated, and flexible health service delivery has now entered the everyday health care lexicon.^33^ However, this switch in the way health professionals work required significant change in policy and funding by both federal and state governments. In March 2020, the Australian Commonwealth Government introduced temporary telehealth and telephone consultation MBS item numbers that enabled the rollout of a universal telehealth model for all Australians.^34^ This program enabled patients to access approved health care via video or telephone consultations from home. Expanded MBS funding made it possible for anyone to consult GPs online or by telephone regardless of postcode. The conversion from face‐to‐face appointments to virtual interactions in support of physical distancing would not have happened so quickly without government intervention. That is, it required government support to finally move the needle.

## 5 RETAIN THE GAINS

The need for face‐to‐face delivery of certain health‐related procedures and tests has not changed—but a willingness to support virtual consultations through the MBS has dramatically increased. Policy change has strengthened the role of telehealth services in Australia's health care industry, marking a more inclusive way to meet patients' basic needs across the nation. However, these changes were only ever meant to last the duration of the crisis, the implementation of which policy‐makers viewed as a short‐term stop gap.^35^ Although the expansion of virtual access was originally implemented as an interim ‘pandemic’ measure, in January 2022 the Australian Commonwealth Government announced it would adopt the MBS Review Taskforce recommendation to embed telehealth as a permanent fixture of the Medicare landscape.^35^ This significant public health reform has paved the way for a more flexible and inclusive universal health care system but, more importantly, takes substantial strides towards obtaining health access parity for rural and remote patients. Yet, policy changes alone will not guarantee these gains. How providers choose to implement telehealth programs and whether or not they consider rural Australians in future delivery models will be essential determinants if urban/rural medical capacity is ever to find an equilibrium.

## 6 CONCLUSION

All Australians, and in particular rural Australians, are well positioned to become long‐term beneficiaries of the COVID‐19‐driven reform of health care delivery models. Thankfully, accessing health care remotely during the ongoing pandemic has become the norm. Subsequent changes to the MBS to permanently include telehealth items for GP consultations is a positive gain but one key question remains: Can the health care system integrate this model of delivery into ‘business as usual’ to ensure that virtual services to rural Australia are sustained after the pandemic?

COVID‐19 has presented an opportunity to retain the gains and embed virtual health solutions as a standard mode of care. Linking‐in virtually to GPs, allied health professionals and medical specialists will offer fast and reliable access to clinical care for people living in the vast, marginalised and underserviced regions outside of metropolitan areas. Enhancing telehealth options for rural and remote Australians can also reduce the travel burden and address place, proximity and mobility challenges for vulnerable patients. There is an opportunity to craft creative telehealth models of care that can complement the skills of understaffed rural clinics and/or better facilitate telehealth partnerships between urban‐based health providers and rural patients. Firmly establishing universal telehealth as a regular and fully funded feature of rural, regional and remote health services is an unexpected, post‐pandemic benefit for rural Australians. An inclusive telehealth policy that is agnostic towards location might also go some way to reducing the high level of urban paternalism that has previously dominated the Australian health system. Therefore, while confidence in virtual models of care is high, it is time for policy‐makers and practitioners alike to make significant reductions in rural health inequity by embedding telehealth for all rural Australians as a permanent fixture in primary health care delivery models.

## AUTHOR CONTRIBUTIONS

LAC: conceptualization; data curation; formal analysis; investigation; writing – original draft. OTM: conceptualization; supervision; writing – review and editing. AWT‐R: conceptualization; supervision; writing – review and editing.

## CONFLICT OF INTEREST

Dr Lisa A. Caffery is deputy chair of the Central Queensland Hospital and Health Service Board. A/Prof Olav T. Muurlink and Prof Andrew W. Taylor‐Robinson have no conflicts of interest or relevant disclosures.

## ETHICS APPROVAL

No ethics approval statement as this is a commentary. Other aspects of this study that involved primary data collection were subject to CQUniversity Human Research Ethics approval (21425).
